# Special Issue “Novel Targeted Therapies and Drugs in Cancer”

**DOI:** 10.3390/ijms27156942

**Published:** 2026-08-02

**Authors:** Maria Teresa Vietri

**Affiliations:** Department of Precision Medicine, University of Campania “Luigi Vanvitelli”, Via L. De Crecchio, 7, 80138 Naples, Italy; mariateresa.vietri@unicampania.it

Over the past decades, major advances have been achieved in the field of anticancer therapies, with several breakthroughs representing true milestones in cancer treatment. Notable examples include the introduction of immunotherapy in renal cancer, melanoma, and lung cancer, as well as cyclin-dependent kinase inhibitors in breast cancer. These therapeutic advances have led to unprecedented improvements not only in patient survival, but also in quality of life outcomes that would have been scarcely imaginable until relatively recently.

Despite this progress, substantial challenges remain. Cancer is an adaptive and mutagenic disease, capable of continuously developing escape mechanisms that ultimately limit the efficacy of therapeutic interventions, particularly in patients with unresectable disease, either upfront or following treatment. As a result, many therapies eventually lose their effectiveness.

Consequently, the identification of novel therapeutic targets and the development of new anticancer agents must remain the central focus of cancer research. This Special Issue brings together three review articles and three original research papers, offering a broad overview that ranges from emerging therapeutic targets, including p21-activated kinases 4–6 (PAK4–6) [contribution 1], isocitrate dehydrogenase 1 and 2 (IDH1/2) [contribution 2], and extracellular vesicles (EVs) [contribution 3] to novel antitumor compounds, such as isorhapontigenin (ISO) [contribution 4] and a chalcone isolated from the plant *Chromolaena tacotana* [contribution 5]. The collection also highlights the highly innovative perspective proposed by Kumar and colleagues, who describe an active genomic adaptation of cancer cells in response to chemotherapy [contribution 6].

Although these studies explore diverse aspects of tumor biology, from signaling pathways to natural compounds and adaptive genomic responses, they converge on a common objective: identifying new vulnerabilities that can be therapeutically exploited in cancer.

Figure 1 summarizes the topics addressed in this Special Issue. On the left are the PAK and IDH pathways, which represent promising targets for the immediate future of anticancer drug research, and extracellular vesicles (EVs), which are explored both as biomarkers and as therapeutic targets. On the right are the two compounds with direct antitumor activity, isorhapontigenin and chalcotanine. At the bottom center is the process of genomic adaptation developed by tumor cells in response to therapy, a mechanism that must be overcome in order to improve the effectiveness of treatments.

Starting from the reviews by Lu et al. [contribution 1] on PAK4–6 inhibitors and Ivanov et al. [contribution 2] on IDH inhibitors, two highly relevant pathways emerge in the search for novel anticancer therapeutic targets.

The p21-activated kinase (PAK) family regulates several cellular processes, including cytoskeleton organization, cell migration, proliferation, and survival. Based on structural and regulatory differences, PAK proteins are divided into two groups: Group I (PAK1–3) and Group II (PAK4–6). Group I PAKs contain an autoinhibitory domain that regulates their activity and are broadly involved in physiological cellular processes, including actin cytoskeleton dynamics, cell migration, proliferative signaling, and normal tissue development such as neuronal and cardiovascular functions. In contrast, Group II PAKs lack this canonical autoinhibitory domain and are more frequently associated with cancer-related processes, including cell survival, tumor invasiveness, regulation of cell motility, and oncogenic signaling. Because of these characteristics, Group II members (particularly PAK4) have been extensively investigated as potential therapeutic targets in cancer [1,2].

Increasing evidence also indicates that PAK signaling may contribute to immune evasion and influence the response to immunotherapy. In particular, PAK activity appears to correlate with tumor microenvironment characteristics and immune cell infiltration. Inhibition of PAK4 has been shown to enhance antitumor immunity and improve the efficacy of PD-1 blockade in preclinical models [3], suggesting that PAK signaling may represent a link between oncogenic pathways and tumor immune regulation.

Despite the strong preclinical rationale for targeting PAK signaling, drug development has proven challenging. PF-3758309, the first potent ATP-competitive pan-PAK inhibitor, showed antitumor activity in preclinical models, but its clinical development was discontinued due to toxicity and unfavorable pharmacokinetics [4]. More recently, molecules such as FRAX486 have shown the ability to reduce metastatic potential in triple-negative breast cancer models [5]. However, the development of clinically effective PAK inhibitors remains difficult because of the high structural similarity among PAK family members and the important physiological roles of these kinases in normal tissues, which raise concerns about selectivity and toxicity.

In contrast, inhibitors targeting isocitrate dehydrogenase (IDH) represent a more advanced stage of clinical development. IDH inhibitors constitute an emerging class of anticancer drugs that have already been introduced into clinical practice for the treatment of acute myeloid leukemia (AML) and are currently being investigated in other malignancies, including gliomas and cholangiocarcinoma (CCA).

IDH is a key metabolic enzyme involved in the tricarboxylic acid (TCA) cycle and plays an essential role in cellular metabolism, oxidative stress regulation, gene expression, and cellular differentiation [6]. Mutations in IDH were initially identified in glioma and colorectal cancer and were later detected in myeloid malignancies, particularly myelodysplastic syndromes and AML [7]. Approximately 15–20% of AML cases harbor IDH mutations.

Three IDH inhibitors are currently approved by the FDA for IDH-mutant AML: *enasidenib* (IDH2 inhibitor), approved in 2017 for relapsed or refractory AML; *ivosidenib* (IDH1 inhibitor), approved in 2019 for both relapsed/refractory AML and newly diagnosed patients ineligible for intensive chemotherapy; and *olutasidenib*, another IDH1 inhibitor approved in 2022 for relapsed or refractory AML after prior therapies. These drugs are generally well tolerated, although adverse effects such as elevated liver enzymes, fatigue, nausea, and QT prolongation may occur [8,9,10].

The clinical potential of IDH inhibition is also expanding beyond hematological malignancies. For example, *vorasidenib*, a brain-penetrant dual IDH1/2 inhibitor, significantly improved progression-free survival in patients with IDH-mutant low-grade gliomas in a phase III trial [11]. IDH mutations are also present in approximately 10% of CCA, particularly intrahepatic tumors. In this setting, the IDH1 inhibitor *ivosidenib* improved progression-free survival in the ClarIDHy trial [12], while other agents such as *olutasidenib* are currently under clinical investigation.

Although substantial progress has been made in understanding IDH-mutant cancers and developing targeted therapies, further research is required to fully clarify the biological complexity of these mutations and to expand the clinical use of IDH inhibitors, particularly in earlier treatment settings.

Compared with signaling pathways directly involved in tumor development, such as PAK and IDH, the biology of extracellular vesicles (EVs) represents an even more complex research field due to their broad distribution and functional heterogeneity. EVs are nanosized particles released by most cell types and mediate intercellular communication through the transfer of bioactive molecules, including proteins, lipids, and nucleic acids. Based on their biogenesis and size, EVs are commonly classified into exosomes, microvesicles, and apoptotic bodies [13,14,15].

Interest in EVs has increased considerably because they may serve both as non-invasive biomarkers and potential therapeutic targets. The molecular components transported by EVs can reflect the physiological or pathological state of their cells of origin and may actively participate in disease progression by modulating inflammation, fibrosis, immune responses, and tumor growth [16]. In hepatobiliary diseases, EVs have been investigated in a wide range of conditions, including viral hepatitis, metabolic dysfunction–associated steatotic liver disease, alcohol-associated liver disease, autoimmune hepatobiliary disorders, and hepatobiliary malignancies.

One of the major challenges in current research is defining how the potential of EVs can be effectively translated into clinical applications. Standardization of EV isolation, characterization, and monitoring methods remains essential to ensure reproducibility and reliable clinical implementation, highlighting the need for further methodological and translational studies [17].

The Special Issue also includes studies focusing on natural compounds with potential anticancer activity. Two original articles by Mendez-Callejas et al. [contribution 5] and Li et al. [contribution 4] investigate the antitumor effects of natural molecules in triple-negative breast cancer (TNBC) and muscle-invasive bladder cancer (MIBC) cell models, respectively.

In the study by Mendez-Callejas et al. [contribution 5], the authors describe the anticancer activity of chalcotanina, a novel chalcone isolated from *Chromolaena tacotana*, a plant endemic to Colombia. Chalcones belong to the flavonoid family and are widely distributed in fruits and vegetables. Historically used in traditional medicine for inflammatory conditions, these compounds have recently attracted attention for their antioxidant and anticancer properties [18]. Due to the limited availability of natural sources, the synthesis of chalcone derivatives has become a common strategy for studying their pharmacological potential [19]. Previous studies have shown that chalcones can exert anticancer effects through multiple mechanisms, including apoptosis induction, modulation of autophagy, cell-cycle disruption, and regulation of inflammatory pathways [20].

The work by Mendez-Callejas and colleagues confirms the antitumor potential of this class of compounds and demonstrates the activity of chalcotanina in several cancer cell lines. The molecule showed selective cytotoxicity in breast, cervical, and prostate cancer cells, with the strongest effect observed in the TNBC cell line and limited toxicity toward normal cells. Mechanistically, chalcotanina promotes apoptosis through activation of caspases-3 and -7 and downregulation of the anti-apoptotic protein XIAP. Additional mechanisms include mitochondrial membrane potential disruption and induction of autophagy. Molecular docking analyses further suggest interactions with key pro-survival proteins such as Bcl-2, Bcl-XL, Mcl-1, mTOR, and XIAP, potentially impairing their protective role in cancer cells.

These findings support further investigation of chalcones, and chalcotanina in particular, as potential anticancer agents, with future studies needed to better define their pharmacokinetic and pharmacodynamic properties and evaluate their translational potential in oncology.

The study by Li et al. [contribution 4] investigates the molecular mechanisms underlying the anticancer effects of isorhapontigenin (ISO) in MIBC. ISO is a stilbene compound isolated from the traditional Chinese medicinal plant *Gnetum cleistostachyum*. Stilbenes are polyphenolic molecules known for their antioxidant, anti-inflammatory, and anticancer properties.

Previous studies have demonstrated that ISO inhibits tumor growth and invasiveness in several malignancies. For example, ISO suppresses bladder cancer progression through the STAT1/FOXO1 signaling axis [21], reduces cancer stem-like properties by downregulating CD44 [22], and impairs cancer cell invasion and autophagy through the Dicer/miR-145/SOX2/miR-365a pathway [23].

Building on these findings, Li and colleagues performed transcriptomic profiling of T24 bladder cancer cells treated with ISO. Their analysis identified 1047 differentially expressed genes (DEGs), including 596 downregulated and 451 upregulated genes. Functional annotation and pathway analyses revealed that ISO treatment significantly affects genes involved in cell migration, invasion, metabolism, proliferation, and angiogenesis. In particular, ISO activates genes related to inflammatory responses while repressing pathways associated with hypoxia signaling, glycolysis, actin cytoskeleton organization, and the tumor microenvironment. These transcriptomic changes suggest that ISO may inhibit tumor progression by remodeling the tumor microenvironment, altering cancer metabolism, and interfering with cellular processes essential for tumor survival and dissemination.

Finally, this Special Issue also highlights an emerging and highly innovative concept in cancer biology: tumor genomic adaptation to therapy. While chemotherapy resistance has traditionally been explained by the selection of pre-existing mutations within genetically heterogeneous tumor populations [24,25,26], recent evidence suggests that resistance may also arise during treatment itself. Kumar et al. [contribution 6] demonstrated that repeated exposure of genetically identical colorectal cancer cells to SN-38, the active metabolite of irinotecan, leads to the progressive accumulation of mutations predominantly in non-coding heterochromatic regions, frequently located at Topoisomerase I (Top1) cleavage sites. DNA breaks induced by the drug and repaired through homologous recombination (HR) and non-homologous end joining (NHEJ) progressively reshape the genome, reducing the number of Top1-sensitive sites and thus the cytotoxic effect of SN-38. Notably, resistant clones remained sensitive to other chemotherapeutic agents such as doxorubicin, highlighting the specificity of this adaptive mechanism.

Together, these findings expand the classical view of chemotherapy resistance, suggesting that therapy-driven genomic adaptation may complement clonal selection and should be considered in future strategies aimed at limiting tumor evolutionary plasticity [6].

In summary, emerging targets such as PAK kinases and mutant IDH enzymes highlight promising directions for the development of new targeted therapies. At the same time, extracellular vesicles are attracting increasing interest as potential biomarkers and therapeutic tools. Natural compounds such as chalcotanina and ISO represent additional strategies that may broaden current anticancer approaches. Finally, the work by Kumar et al. on irinotecan resistance introduces the innovative concept of tumor genomic adaptation to therapy, opening new perspectives for combination approaches aimed at overcoming drug resistance.

Taken together, the studies included in this Special Issue, although addressing diverse therapeutic targets, reflect a common conceptual shift in oncology: a transition from single-pathway targeting toward a broader exploitation of tumor-specific biological vulnerabilities. Rather than focusing solely on predefined druggable genes, these contributions highlight therapeutic opportunities emerging from tumor biology itself, including genomic instability, immune-related mechanisms, and metabolic adaptations. Embracing this biological complexity will be essential to address tumor heterogeneity and therapeutic resistance, ultimately enabling more precise and durable treatment strategies.

## Figures and Tables

**Figure 1:**
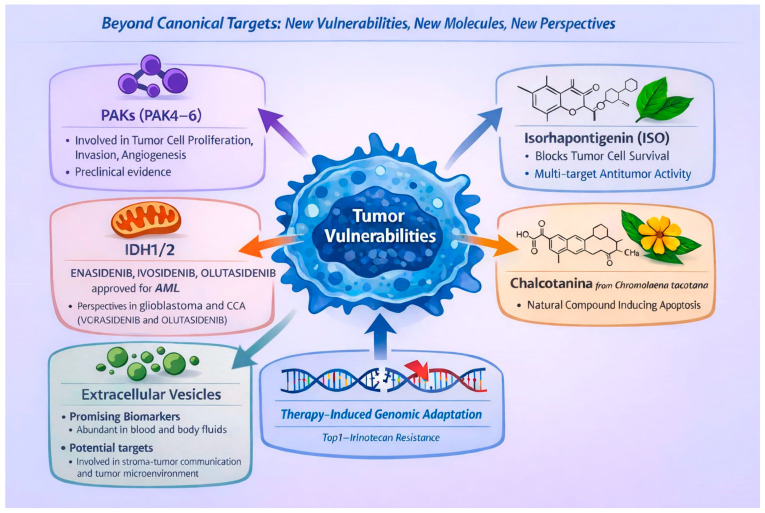
Main topics addressed in this Special Issue. On the left, emerging molecular targets: the PAK and IDH pathways and extracellular vesicles (EVs), investigated as biomarkers and potential therapeutic targets. On the right, two natural compounds, isorhapontigenin (ISO) and chalcotanina. At the bottom, tumor genomic adaptation as a mechanism of resistance to irinotecan.
